# Image of the Month: Enterocolitis Following a Pull-through for Total Colonic Hirschsprung Disease in a 2-Year-Old Boy

**DOI:** 10.1055/s-0040-1721050

**Published:** 2021-01-09

**Authors:** Anisha Apte, Elise McKenna, Marc A. Levitt

**Affiliations:** 1 Department of Surgery, The George Washington University School of Medicine and Health Sciences, Washington, District of Columbia, United States; 2 Department of General and Thoracic Surgery, Children's National Medical Center, Washington, District of Columbia, United States; 3 Division of Colorectal and Pelvic Reconstruction Surgery, Children's National Medical Center, Washington, District of Columbia, United States

**Keywords:** Hirschsprung disease, enterocolitis, pull-through

## Abstract

We present a case of a 2-year-old boy with total colonic Hirschsprung disease (HD) who underwent an ileostomy as a newborn, and then colectomy and pull-through at 10 months of age. Since then he has presented four times with enterocolitis. The case is presented with a focus on evaluating patients with HD who present with obstructive symptoms following corrective surgery. A key image is presented along with questions formatted as a quiz to guide readers through critically evaluating the case.

## Case Report

**Fig. 1:**
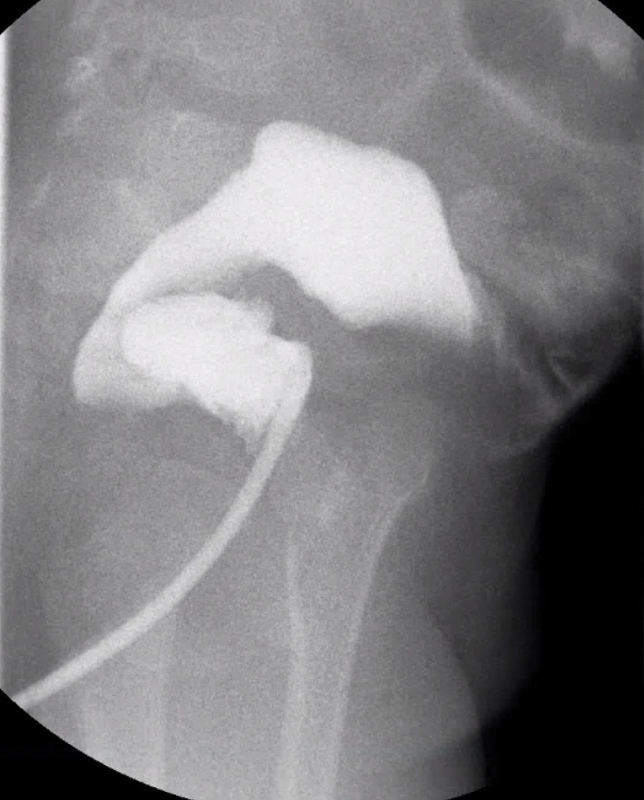
A contrast enema study.

You are presented with a 2-year-old boy with a history of Hirschsprung disease (HD) who has previously undergone a newborn ileostomy, and then a colectomy and pull-through at 10 months of age, and has subsequently had four episodes of enterocolitis. You review a contrast enema study that was completed after the patient recovered from the enterocolitis ( Fig. 1 ).

## Discussion

Hirschsprung associated enterocolitis (HAEC) is a feared complication in patients with HD, which typically presents as abdominal distension, fever, and diarrhea. 1 It can occur both prior to and after HD surgery and can develop when there is stasis, bacterial overgrowth, and bacterial translocation. Although the exact physiology of HAEC is unknown, causes for obstruction should be routinely evaluated in patients who have undergone corrective surgery and thereafter have recurrent HAEC.

Obstructive symptoms in HD patients post pull-through have been reported between 8 and 30% postoperatively, and HAEC has been reported between 25 and 37%. 2 There are five potential explanations for persistent obstructive symptoms after pull-through:

- Mechanical obstruction
- Aganglionosis/transition zone pull-through
- Internal sphincter achalasia (absent rectoanal inhibitory reflex [rair] causing obstructive symptoms)
- Motility disorder
- Functional megacolon 3

Initial evaluation with a contrast enema and an examination under anesthesia can help identify the presence of a mechanical obstruction. 1 4 The contrast study provides the answer to the anatomic problem with this case's pull-through. It is likely that the recurrent enterocolitis episodes are due to mechanical obstruction caused by a Duhamel spur. We will discuss the Duhamel spur in addition to the other common causes of mechanical obstruction.

**Fig. 2:**
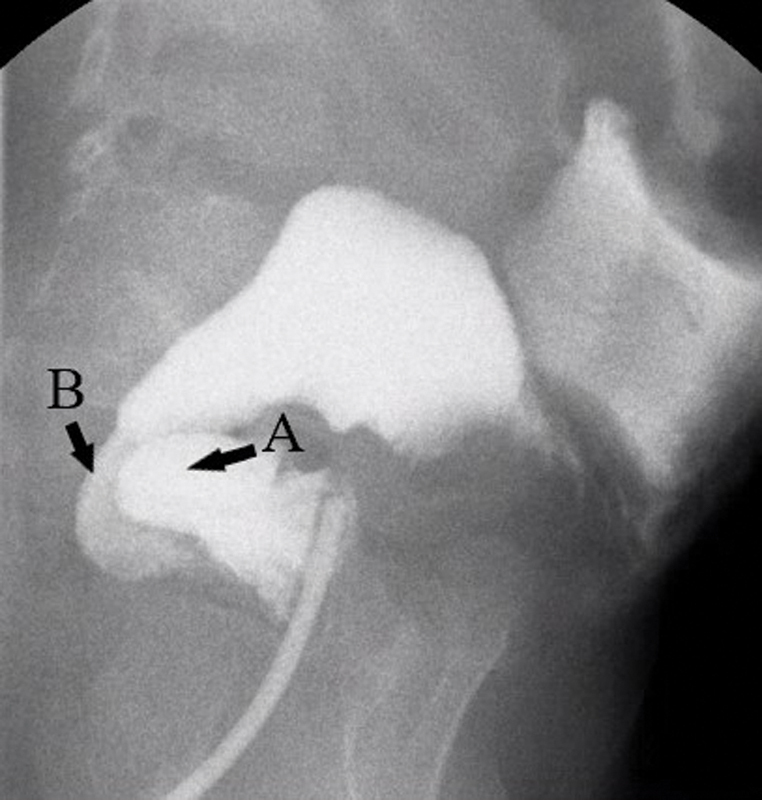
Contrast enema study showing Duhamel spur. ( **A** ) Aganglionic pouch. ( **B** ) Ileum pull-through segment.

In the case of this 2-year-old boy, the contrast enema shows what appears to be his rectal pouch compressing the bowel just proximal to it ( Fig. 2 ). At 10 months of age, this boy underwent colectomy and ileo-Duhamel pull-through; however, the Duhamel pouch and pulled-through ileum were not adequately united into a single lumen, leading to a Duhamel spur. Accumulation of stool in the pouch eventually causes compression and obstruction of the ileum, leading to stasis and recurrent enterocolitis. The treatment is to remove the spur. This can be done with an endovascular stapler placed transanally. In this case, the staple line was extended, thus removing the spur, and the patient began to stool better and has not had another episode of enterocolitis. If this maneuver is not possible or does not improve symptoms, then resection of the pouch and a redo operation with conversion to ileo-anal anastomosis is in order. 3 5

Other mechanical issues that can be wrong with an HD pull-through include an obstructing seromuscular cuff, an anastomotic stricture, and a twist of the pull-through. 6 An obstructing seromuscular cuff is a complication from a Soave procedure and occurs when the residual aganglionic sleeve of the intentionally preserved muscular layer causes narrowing around the pull-through. This is thought to occur due to a constant contracted state of the aganglionic muscle 7 and may occur if the cuff is not split, is not split adequately, or rolls up. To reduce the occurrence of this, many surgeons who perform the Soave pull-through have modified it so that the amount of residual cuff is limited to just 1 to 2 cm from the beginning of their dissection. 7 One could affectionately refer to this modification as a “Soave-son” as it is approaching the Swenson technique. A long obstructing cuff can sometimes be treated with intraperitoneal division of the obstructing cuff using a laparoscopic approach. 6 Such an approach is most relevant for very long cuffs, or cuffs that were created during a transabdominal Soave procedure, rather than one done transanally. A redo transanal pull-through with removal of the muscular cuff and any proximally dilated colon is also an effective strategy. 7 In the case of anastomotic stricture, these can usually be addressed with serial dilations although if unsuccessful, resection, and redo pull-through may also be required. 3 A twisted pull-through requires a redo. In addition to mechanical problems with a pull-through, the distal aspect may be in transition zone which leads to obstructive symptoms and requires a reoperation. 8

If no anatomic or pathologic problem is identified, a nonrelaxing motility disorder—even with an anatomically and pathologically correct pull-through—is a rare occurrence and is best evaluated by colonic manometry. A functional megacolon should respond to laxative therapy.

## Conclusion

Mechanical obstruction is common after a pull-through procedure in patients with HD. An anatomic or pathologic cause for obstruction can lead to recurrent enterocolitis, as well as other obstructive symptoms such as chronic abdominal distension and failure to thrive. These patients and should be evaluated with a contrast enema and an examination under anesthesia. It is important for the surgeon to recognize that different complications exist depending on the pull-through procedure that was initially performed and warrant different surgical approaches in management. Although some less invasive techniques exist to address these issues, patients may require a redo of their pull-through to resolve the obstructive symptoms.

## Notes

New Insights and the Importance for the Pediatric Surgeon Complications after a pull-through procedure vary depending on the type of operation done, and may cause obstructive symptoms, including enteritis. All patients presenting with obstructive symptoms after a pull-through procedure should be evaluated with a contrast enema and an examination under anesthesia.
